# Prevalence and Factors Associated with Vitamin D Deficiency and Hyperparathyroidism in HIV-Infected Patients Treated in Barcelona

**DOI:** 10.5402/2012/485307

**Published:** 2012-07-12

**Authors:** Elisabet Lerma, M. Ema Molas, M. Milagro Montero, Ana Guelar, Alicia González, Judith Villar, Adolf Diez, Hernando Knobel

**Affiliations:** Department of Internal Medicine and Infectious Diseases, Hospital del Mar, 08003 Barcelona, Spain

## Abstract

Vitamin D deficiency is an important problem in patients with chronic conditions including those with human immunodeficiency virus (HIV) infection. The aim of this cross-sectional study was to identify the prevalence and factors associated with vitamin D deficiency and hyperparathyroidism in HIV patients attended in Barcelona. Cholecalciferol (25OH vitamin D3) and PTH levels were measured. Vitamin D insufficiency was defined as 25(OH) D < 20 ng/mL and deficiency as <12 ng/mL. Hyperparathyroidism was defined as PTH levels >65 pg/mL. Cases with chronic kidney failure, liver disease, treatments or conditions potentially affecting bone metabolism were excluded. Among the 566 patients included, 56.4% were exposed to tenofovir. Vitamin D insufficiency was found in 71.2% and 39.6% of those had deficiency. PTH was measured in 228 subjects, and 86 of them (37.7%) showed high levels. Adjusted predictors of vitamin D deficiency were nonwhite race and psychiatric comorbidity, while lipoatrophy was a protective factor. Independent risk factors of hyperparathyroidism were vitamin D < 12 ng/mL (OR: 2.14, CI 95%: 1.19–3.82, *P*: 0.01) and tenofovir exposure (OR: 3.55, CI 95%: 1.62–7.7, *P*: 0.002). High prevalence of vitamin deficiency and hyperparathyroidism was found in an area with high annual solar exposure.

## 1. Introduction

 Vitamin D is a steroid liposoluble hormone and can be made available to the individuals in two forms. First, vitamin D3 or cholecalciferol is synthesized in the skin in response to ultraviolet B radiation and is present in oil-rich fish (salmon, mackerel, and herring), egg yolks, and liver [1]. Second, vitamin D2 or ergocalciferol is obtained from the UV irradiation of the yeast sterol ergosterol and is found in sun-exposed mushrooms [2]. Both vitamins undergo identical metabolism, and as they are biologically inert, they require two hydroxylations, in the liver and in the kidney, to become 1,25dihydroxyvitamin D [1,25(OH)_2_D] which is the biologically active form of the hormone [3, 4]. In the intestine this stimulates the absorption of calcium and phosphorus, and it helps to regulate the metabolism of both minerals within bone and kidney interaction [5]. Assessment of vitamin D is based on measurement of serum 25(OH)D [6, 7] that is the most stable and plentiful metabolite of vitamin D in serum and has a half-life about 21 days [8]. 

The main source of vitamin D is exposure to sunlight [9, 10]. Therefore, an insufficient exposure to sunlight is a major cause of vitamin D deficiency. Other causes of vitamin deficiency are sunscreen sun protection [2], dark skin body mass index (BMI) greater than 30 [11, 12], malabsorptive conditions, and use of a wide variety of medications including antiretroviral drugs. Vitamin D levels are also related to seasonal fluctuation, geographic latitude, time of the day for sunlight exposure, and age.

Vitamin D deficiency results in abnormalities in bone metabolism [13, 14] and in calcium and phosphorus homeostasis [15, 16] and is increasingly recognized as a key factor in many chronic diseases [17, 18].

HIV patients suffer from several risk factors of vitamin D deficiency [19–21]. Not only they have a high prevalence of disorders related to lower vitamin D levels [22], but also they are exposed to some antiretrovirals that can lead to vitamin D deficiency [23], specially efavirenz [24–26] and protease inhibitor, which seem to be associated with higher rates of vitamin D deficiency. Moreover, some studies suggest a protective effect of vitamin D against HIV disease progression, mortality, and AIDS events that might be explained by its role in immune function [27]. 

## 2. Methods

### 2.1. Study Design, Setting, and Participants

This is a cross-sectional study of 566 HIV-infected patients, older than 18 years old, with and without antiretroviral treatment, recruited in a consecutive manner from August 2010 to August 2011, in a tertiary referral center for HIV patients in Barcelona, Spain.

Data were collected every 3 to 6 months and included CD4 cell counts, viral loads (COBAS, AmpliPrep/TaqMan HIV-1 test, Roche Diagnostics), HIV-related parameters including time since HIV diagnosis, CDC stage, cART regimen, medical history, hepatitis coinfection, ethnicity, and patients demographics. The primary outcomes were plasma levels of 25(OH)D (competitive chemiluminescent immunoassay, CLIA, City, Country) and intact PTH (solid-phase sequential chemiluminescent immunoassay, Manufacturer, City, Country). Secondary variables included calcium, phosphorus, cholesterol, and renal and liver parameters. 

Patients with kidney insufficiency (defined as GFR < 30 mL/min) and liver insufficiency (Child Pugh Stage C) and those patients whose records contained insufficient data were excluded from the analysis.

### 2.2. Definitions

Vitamin D insufficiency was defined as 25(OH)D <20 ng/mL and deficiency as <12 ng/mL. Hyperparathyroidism was defined as PTH levels >65 pg/mL. Undetectable viral load (HIV RNA) was defined as HIV RNA <20 copies/mL.

### 2.3. Statistical Analysis

Proportions were compared with *χ* ^2^ and Fisher exact test.

Quantitative data were analyzed using Student's *t*-test for normal variables. The Mann Whitney *U* test was used to compare variables with nonnormal distribution. The variables observed as risk factor of vitamin D deficiency or hyperparathyroidism in the univariate analysis were included in the logistic regression model (SPSS version 18 was used to all statistics analysis).

## 3. Results

### 3.1. Demographic and Baseline Information

A total of 592 patients were eligible for the study. From those, participants suitable for inclusion because the records had the necessary information were 566.

Among the participants, 400 (70.7%) were men and 480 (84.8%) were Caucasian (86 from different races), with a median age of 46 years (DS 9.5). The predominant mode for HIV transmission was IDU in 291 cases (51%). The median nadir CD4 cell count was 156 (49–269) cells/mm^3^, and current CD4 was 537 cell/mm^3^. A total of 492 patients (87%) had undetectable viral load. At the time of sampling, 549 patients were taking cART, with a median exposure time of 8.9 years (4.9–11.7). From those, 319 (56.4%) were exposed to tenofovir, 115 (20.3%) to abacavir, and 453 (80%) to ritonavir-boosted protease inhibitor regimen.

### 3.2. Vitamin D and PTH: Predictor Factors

In the series 403 (71.2%), patients showed plasma levels of vitamin D in the range of insufficiency and 244 (39.6%) within deficiency levels. Of the 566 patients enrolled in the trial, PTH was measured in 228 cases, with high levels (>65 ng/mL) in 86 of them (37.7%). The median PTH in all patients with hyperparathyroidism was 90 pg/ml (range: 66–200).

Predictors of vitamin D deficiency were (Table 1) non-Caucasian race, OR: 3.18 (CI 95%: 1.49–6.78; *P*: 0.003), and psychiatric concomitant disorders OR: 1.5 (CI 95%: 1.03–2.18; *P*: 0.003), while lipoatrophy was a protective factor OR 0.67 (CI 95%: 0.46–0.99; *P*: 0.05). Independent risk factors for predicting levels of PTH > 65 pg/mL were (Table 2) vitamin D plasma levels <12 ng/mL (OR: 2.14. IC95%: 1.19–3.82, *P*: 0.01) and tenofovir exposure (OR : 3.55, IC95% 1.62–7.7, *P*: 0.002). Elevated PTH levels were associated with the use of tenofovir regardless of the vitamin D level, with a significant difference compared to patients with no exposure to this drug (Table 3). Those patients with elevated PTH receiving tenofovir present with estimated glomerular filtration rates >60 ml/min as well as normal serum calcium and phosphorus levels. Age, gender, BMI, dyslipidemia, diabetes, hepatitis B or C coinfection, current CD4 cell count, current HIV-1 RNA load, time of exposure to ART, and use of protease inhibitors were not associated with abnormal vitamin D or PTH levels.

## 4. Discussion

### 4.1. Vitamin D is Recognized as a Key Factor for Many Chronic Diseases

Therefore, it seems plausible that vitamin D screening and the use of this hormone supplementation could benefit HIV patients when beginning ART. 

The present study showed a high prevalence of vitamin D deficiency in HIV patients in an area with high solar exposure. 

The prevalence of vitamin D suboptimal levels among HIV patients goes from 60 to 95% (including those with insufficiency and deficiency). In EUROSIDA [28] cohorts the prevalence is of 83%, while in two cohorts in Swiss [29] and Denmark [30], respectively, it represents a 42% and 95%. Regardless, there are two different studies in the United States, where despite having a 63% to 75% of vitamin D deficiency among HIV-infected people, the prevalence was lower than in uninfected HIV people [22, 31]. It is difficult to compare the prevalence of vitamin D insufficiency and deficiency because different definitions were used. Some studies define vitamin D deficiency as a 25OH D less than 20 ng/mL, and insufficiency as a 25OH D of 21–29 ng/mL [32, 33]. However, in cohorts of HIV patients, such as EuroSIDa, insufficiency is defined as 25OH D levels below 20 ng/mL and deficiency as levels less than 12 ng/mL [28]. The vitamin D deficiency was observed regardless of age, sex, BMI, dyslipidemia, diabetes, hepatitis B or C coinfection, current CD4 cell count, current HIV-1 RNA load, time of exposure to ART, and use of protease inhibitors.

Race, seasonality, overweight, nadir CD4 cell count less than 200 cell/mm^3^, and exposure to some ART, specially efavirenz, have been identified in other studies as risk factors for vitamin D deficiency [34, 35].

In our study, predictors of vitamin D deficiency were being non-Caucasian OR: 3.18 (CI 95%: 1.49–6.78, *P*: 0.003) and having psychiatric comorbidity OR: 1.5 (CI 95%: 1.03–2.18; *P*: 0.003), while lipoatrophy was a protective factor OR 0.67 (CI 95%: 0.46–0.99; *P*: 0.05). 

Black ethnic was associated with vitamin D deficiency, because they require a longer exposure to produce the same amount of vitamin D than white people do [36]. Patients with psychiatric disorders are also identified as patients with a higher risk for vitamin D deficiency, as poorest diets and a lack of exposure to sunlight are common in this kind of patients [37]. Both factors play a major role in the development of vitamin D deficiency [38, 39]. 

We identified lipoatrophy as a protective factor. This could be explained by the fact that vitamin D is a liposoluble hormone, so lipoatrophy could avoid the accumulation of this vitamin in the body fat, increasing circulating levels. Some studies do identify lipoatrophy as a risk factor for vitamin D deficiency [40] against other studies where lipoatrophy is not related with vitamin D [41].

Independent risk factors for high levels of PTH were vitamin D < 12 ng/mL and tenofovir exposure. Elevated PTH levels were associated with the use of tenofovir regardless of the values of vitamin D, with a significant difference compared to patients with no exposure to tenofovir. Cross-sectional data have linked tenofovir with higher parathyroid hormone (PTH) concentration in patients with vitamin D deficiency [42]. Masiá et al. [43] performed a longitudinal study to evaluate sequential changes in PTH and 25-hydroxyvitamin D [25(OH) D] levels in patients starting cART with either tenofovir/emtricitabine or abacavir/lamivudine. Tenofovir/emtricitabine exposure was an independent predictor of high PTH levels (≥53 ng/liter). The underlying mechanisms of this effect are not well understood, but it is presumable that tenofovir causes a decrease in intestinal calcium absorption, leading to an increase in PTH secretion. Moreover, vitamin D supplementation has shown a decrease in PTH levels, regardless of baseline levels of vitamin D [44].

Limitations of the present study are mainly related to the observational design and cross-sectional nature of the current analyses. In this respect, the results reported herein are only associations from which no conclusions regarding causality can be drawn. The 25(OH) D measurements were done along the whole year, using the highest value as the referent value in each patient, independently of seasonallity.

Due to the wide range of biological actions of vitamin D and the fact that its deficiency has been associated with many adverse outcomes, such as infectious diseases, muscle weakness, metabolic, and cardiovascular and bone disease, we suggest the importance of screening in HIV patients in whom some of these pathologies are more prevalent and, in accordance with the results, starting a systematic supplementation given the high prevalence of the problem in HIV patients.

## Figures and Tables

**Table 1:** Predictors of vitamin D deficiency (Vit D < 12 ng/mL).

	Univariate analysis		Adjusted analysis	
	OR (IC95%)	*P*	OR (IC95%)	*P*
Non-Caucasian	3.52 (1.69–7.3)	0.0001	3.18 (1.49–6.78)	0.003
Psychiatric comorbidity	1.48 (1.02–2.15)	0.004	1.5 (1.03–2.18)	0.003
Lipoatrophy	0.68 (0.46–1)	0.05	0.67 (0.46–0.99)	0.05

**Table 2:** Risk factors for high levels of PTH (PTH > 65 pg/mL).

	Univariate analysis		Adjusted analysis	
	OR (IC95%)	*P*	OR (IC95%)	*P*
Tenofovir exposure	3.35 (1.86–6.02)	0.0001	3.55 (1.62–7.7)	0.002
Vitamin D < 12 ng/mL	2.2 (1.26–3.82)	0.007	2.14 (1.19–3.82)	0.01
Abacavir exposure	0.48 (0.23–0.98)	0.04	0.87 (0.33–2.31)	0.7

**Table 3:** Interactions between tenofovir exposure and vitamin D deficiency with PTH levels.

	Vitamin D < 12 ng/ml		Vitamin D > 12 ng/ml		*P*	*P*
	Tenofovir	No tenofovir	Tenofovir	No tenofovir	A versus B	A versus C
	(*n*: 52) A	(*n*: 33) B	(*n*: 78) C	(*n*: 65) D		
PTH > 65 pg/mL	31 (59.6%)	11 (33.3%)	33 (42.3%)	11 (16.9%)	*P*: 0.002	*P*: 0.07
Median PTH pg/mL (IQR)	76 (52.5–105.5)	47 (31.5–75)	52 (35–90)	40 (26–57)	*P*: 0.001	*P*: 0.007
